# Acridine Orange and Flow Cytometry: Which Is Better to Measure the Effect of Varicocele on Sperm DNA Integrity?

**DOI:** 10.1155/2015/814150

**Published:** 2015-11-22

**Authors:** Essam-Elden M. Mohammed, Eman Mosad, Asmaa M. Zahran, Diaa A. Hameed, Emad A. Taha, Mohamed A. Mohamed

**Affiliations:** ^1^Dermatology and Andrology Department, Faculty of Medicine, Al-Azhar University, Assiut, Egypt; ^2^Clinical Pathology Department, South Egypt Cancer Institute, Assiut University, Assiut, Egypt; ^3^Urology Department, Faculty of Medicine, Assiut University, Assiut, Egypt; ^4^Dermatology and Andrology Department, Faculty of Medicine, Assiut University, Assiut, Egypt; ^5^Gynaecology and Obstetric Department, Faculty of Medicine, Al-Azhar University, Assiut, Egypt

## Abstract

We evaluated the effect of varicocelectomy on semen parameters and levels of sperm DNA damage in infertile men. A total of 75 infertile men with varicocele and 40 fertile men (controls) were included in this study. Semen analysis and sperm DNA damage expressed as the DNA fragmentation index using acridine orange staining and chromatin condensation test by flow cytometry were assessed before and 6 months after varicocelectomy. The patients were also followed up for 1 year for pregnancy outcome. Semen parameters were significantly lower in varicocele patients compared to controls (*P* < 0.05). Mean percentages of sperm DNA fragmentation and sperm DNA chromatin condensation in patients were significantly higher than those in controls (*P* < 0.05). After varicocelectomy, sperm DNA fragmentation improved significantly, whereas sperm chromatin condensation was not significantly changed. In 15 out of 75 varicocele patients, clinical pregnancy was diagnosed; those with positive pregnancy outcome had significant improvement in sperm count, progressive sperm motility, and sperm DNA fragmentation, but there was no significant difference in sperm DNA condensation compared to negative pregnancy outcome patients. We concluded from this study that acridine orange stain is more reliable method than flow cytometry in the evaluation of sperm DNA integrity after varicocelectomy.

## 1. Introduction

Sperm DNA integrity is important for the transmission of genetic code, and it is considered as a marker of integrity of spermatogenesis and male fertility potential [1]. About 10% of the spermatozoa from fertile men and 20–25% of the spermatozoa from infertile men have measurable levels of DNA damage [2]. High levels of sperm DNA fragmentation (DFI) have been significantly associated with a bad pregnancy outcome [3–5].

Sperm DNA damage may be associated with many environmental conditions such as some medications, pollution, smoking, pesticides, chemicals, high temperature, and various pathologic cases such as cryptorchidism, fever, aging, infection, chemotherapy, cancer, and varicocele [6, 7].

The prognostic value of sperm DNA fragmentation is becoming better than the routine semen parameters, although the cut-off values of it are not established yet [8].

In this study, we evaluate the effect of varicocele on semen parameters and levels of sperm DNA integrity in infertile men with varicocele before and after varicocelectomy by acridine orange staining and flow cytometry.

## 2. Materials and Methods

From January 2012 to March 2015, a total of 75 men with at least 1-year history of infertility, a palpable varicocele, oligo, atheno, or teratozoospermia were selected from our andrology clinic. After the ethical committee approval, all the patients accepted to participate in the study and signed an informed consent. Forty healthy fertile volunteers (control group) were also included in this prospective study.

Patients were subjected to complete history taking and thorough general and local examination. Varicocele was detected clinically and confirmed by scrotal ultrasound (Fukuda Denshi Tellus UF-850XTD, Tokyo, Japan) equipped with color flow imaging when at least 1 scrotal vein had a maximum diameter of at least 3 mm and retrograde flow was observed at rest or after Valsalva maneuver. Grade 1 varicocele was diagnosed when reflux was measured at less than 1 second, grade II was diagnosed when reflux lasted 1-2 seconds, and grade III was diagnosed when reflux was noted at more than 2 seconds as described by Cornud et al. [9].

Semen samples were obtained by masturbation and collected in a sterile plastic container before and 3 months after subinguinal varicocelectomy with loop magnification that was done by either of the 3 surgeons with at least 7 years of experience. They were allowed to liquefy for 30 min at 37°C, after which an analysis was performed to measure the following parameters: sperm concentration/mL, percentage of sperm motility, percentage of abnormal sperm morphology evaluated according to WHO guidelines [10].

### 2.1. Acridine Orange (AO) Assay

The AO assay measures the ability of sperm nuclear DNA to denature by acid which forms metachromatic shift of AO fluorescence from green (native DNA) to red (denatured DNA). The fluorochrome AO intercalates in double-stranded DNA as a monomer which binds to single-stranded DNA. The monomeric AO bound to native DNA fluoresce green, whereas the aggregated AO on denatured DNA fluoresces red [11].

**Figure 1:**
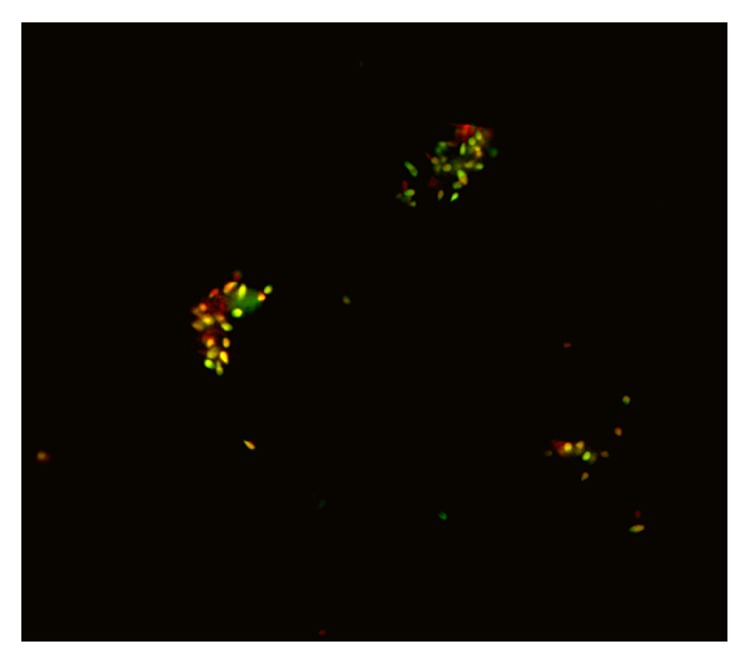
Fluorescence microscopy of acridine orange destained cells shows that spermatozoa displaying green fluorescence were considered to be with normal DNA content, whereas sperms displaying a spectrum of yellow-orange to red fluorescence were considered to be with damaged DNA. Original magnification ×200.

The AO assay may be used for fluorescence microscopy or by flow cytometry. To perform this assay for fluorescent microscopy, thick semen layers are fixed in fixative (methanol : acetic acid 3 : 1) for 2 hours. The slides are stained for 5 minutes and rinsed with water. The slides were washed with distilled water then covered with glass cover and examined under a ZEISS mot plus (Germany) fluorescent microscope at the excitation wavelength of 450–490 nm. An average of 200 sperm cells was evaluated on each slide by the same examiner. Spermatozoa which show green fluorescence were considered as normal DNA content, whereas sperms displaying a spectrum of yellow-orange to red fluorescence were considered to be with damaged DNA (Figure 1). The ratio between (yellow to red)/(green+yellow to red) fluorescence was considered as DFI percentages and the percentage of sample showing a ratio <1 was calculated in the group.

### 2.2. Flow Cytometric Detection of Sperm DNA Chromatin Condensation

**Figure 2:**
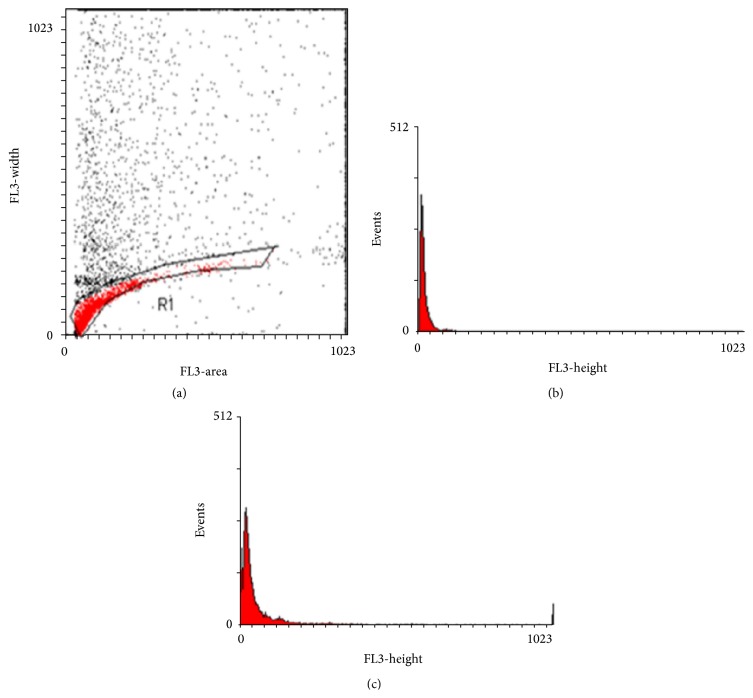
Flow cytometric detection of sperm DNA chromatin condensation. (a) Dot plot histogram (FL3A versus FL3W) obtained after propidium iodide staining of spermatozoa. Cells gated in R1 region were analyzed, while debris and aggregates were excluded from the analysis. The fluorescence intensity of cells (spermatozoa) was measured in R1 gate. (b) Histogram of fluorescence intensity of spermatozoa from normal control person. (c) Histogram of fluorescence intensity of spermatozoa from infertile person with varicocele.

Flow cytometric detection of sperm DNA chromatin damage was made according to the method as described by Martinez-Soto et al. [12]. It depends on the fluorescence emission from sperm cells stained with propidium iodide (PI) that binds to DNA. The semen sample were diluted with phosphate buffered saline (PBS) to 2 × 10^6^ sperm/mL. Fifty *μ*L of semen sample was directly stained with 50 *μ*g/mL PI, using the cycle test kit (Becton Dickinson, USA); PI was mixed with the semen and analyzed immediately by FACSCalibur flow cytometry with CellQuest software (Becton Dickinson Biosciences, USA). Ten thousand events were measured for each specimen; this permitted state of condensation of the sperm chromatin was analyzed, as the DNA condensation is directly related to PI uptake. The geometric mean fluorescence intensity (GMFI) was used to measure the degree of sperm DNA staining with PI. The sperm with altered nuclear condensation (DNA decondensation and fragmentation) takes more stains (Figure 2). These tests were performed on patients before and 3 months after varicocelectomy.

## 3. Statistical Analysis

The SPSS program, version 16.0.1 (SPSS Inc., Chicago, IL), was used in statistical analysis. The data were expressed as mean ± SE and the differences were evaluated by paired *t*-test. Relationships between values were studied by Spearman correlation test. *P* < 0.05 was set as statistically significant.

## 4. Results

Varicocele was detected by physical examination and confirmed by Doppler ultrasound in the 75 patients who entered the study. The mean age was 31 years (range: 20–57) for patients and 30.2 years (range: 21–37) for controls. The main sperm characteristics of control and patients are shown in Table 1. All patients had an abnormality in one or more of these parameters. There was a significant decrease in sperm concentration and percentage of progressive sperm motility and normal sperm morphology in varicocele patients compared to the controls (Table 1).

Comparisons of DFI results and GMFI between controls and patients with varicocele are shown in Table 1. The mean values for DFI and GMFI of sperm DNA chromatin condensation in the control group were lower.

In patients with varicocele, DFI and GMFI had no correlation with sperm concentration, but they were negatively correlated with progressive motility and normal sperm morphology.

Sperm DNA fragmentation by acridine orange and sperm count changed significantly after varicocelectomy, but no significant changes were detected regarding sperm motility, morphology, and DNA chromatin condensation by flow cytometry (Table 2). In 15 out of 75 varicocele patients (20%), clinical pregnancy (confirmed by ultrasound detection of fetal pulse) was achieved. Those with positive pregnancy outcome showed significant improvement in sperm count and sperm motility compared with negative pregnancy group (*P* < 0.05). Also, those with positive pregnancy outcome had significantly lower DNA fragmentation % by acridine orange, but there is no significant difference in sperm DNA condensation by flow cytometry on comparing them to others who failed to make their partners conceive (Table 3).

## 5. Discussion

As it is well known [13–15], our study showed that the mean semen parameters were significantly lower in varicocele patients than in control group. The low sperm count may be due to the high apoptosis in the germ cell that occurs in varicocele, while the decreased motility may be due to the abnormal morphology of the sperm which affects its motility, the increased oxygen concentration of free radicals, or the antisperm antibodies [15].

Also, a large scale study by the WHO showed that infertile men with varicocele have lower sperm concentration significantly compared to idiopathic infertility, but it did not give any evidence regarding motility and morphology of the sperm [16]. Poor chromatin condensation has been correlated with numerous reproductive outcomes [17, 18]. So DFI provides additional information about sperm quality and conception outcome [18, 19].

In our study, there was a high percentage of sperm with damaged DNA among patients with varicocele detected by AO and flow cytometry; this was well documented in the previous studies [20, 21]. Having a higher percentage of sperm with damaged DNA assessed by AO staining suggests that increased DFI is one of the possible causes of infertility and can contribute to the higher prevalence of infertility among patients with varicocele [15, 22].

Several factors associated with varicocele may lead to DNA damage including heat, stress [23], exposure to toxic agents [24], testicular hypoxia [25], androgen deprivation [26], and increased oxidative stress [27].

The high percentage of sperm DFI in varicocele patients may be caused by an impaired chromatin condensation [28] Also, the nature of the nuclear damage could be related to a strong and prolonged exposure to DNA nuclear-damaging factors. Not only the DNA, but also the proteins of the nuclear matrix could lead to an advanced lytic stage [27].

Our results demonstrated that those with positive pregnancy outcome after varicocelectomy had significantly lower sperm DFI with acridine orange test than others who failed. A prospective study of infertile, oligospermic men with clinical varicocele who underwent surgical repair showed a significant improvement in postvaricocelectomy semen parameters and DFI [29]. A lower DFI was associated with higher spontaneous and ART pregnancy rates. Similarly, a positive effect of subinguinal microsurgical clinical varicocele repair on sperm DNA integrity and chromatin compaction (reflected by a significant decrease in sperm DFI and high DNA stainability, resp.) was reported in another prospective study [19]. In a study by [7, 18], the probability of fertilization in natural conception and in intrauterine insemination was found to be close to zero if the proportion of sperm cells with DNA damage exceeds 30% as detected by SCSA proposing the threshold for DFI >30% in infertile men for poor ART outcome.

In our study, we found that after varicocelectomy sperm count improved significantly, while the morphology and motility improvements did not reach statistical significance. Likewise, sperm DNA fragmentation by acridine orange not only improved significantly after varicocelectomy, but also correlated with positive pregnancy outcome, while the improvement measured by chromatin condensation by flow cytometry neither reached statistical significance nor correlated with positive pregnancy outcome.

After varicocelectomy, flow cytometry showed that the DFI was 22.5% of sperm DNA, which may be an accepted threshold for a successful ART outcome. This is comparable to the work of Pasqualotto et al. [5] who reported (27–30%) DNA damage as a threshold for successful pregnancy.

In conclusion, the current study demonstrated an increase in sperm DFI in infertile patients with varicocele. After varicocelectomy, acridine orange test yielded significant results compared to flow cytometry that correlated with pregnancy outcome which suggests that it may be used as cheap and simple DNA integrity evaluation test for diagnostic and prognostic purpose in basic andrology laboratories.

## Figures and Tables

**Table 1:** Clinical and laboratory data of studied groups (mean ± SD).

Variables	Varicocele group (*n* = 75)	Fertile group (*n* = 40)	*P* value
Sperm count (mil/mL)	26.2 ± 2.7^*∗*^	74.2 ± 19.2	0.005
Motility A + B (%)	27.8 ± 16.5^*∗*^	62.5 ± 11.9	0.02
Normal forms (%)	59.8 ± 13.6^*∗*^	71.03 ± 8.2	0.02
Sperm DFI (%) by acridine orange	32.4 ± 7.4^*∗*^	18.2 ± 4.8	0.003
GMFI of sperm DNA chromatin condensation by flow cytometry (%)	25.4 ± 8.8^*∗*^	12.8 ± 2.2	0.005
Mean age (years)	31 ± 8.2	30.2 ± 2	NS

^*∗*^Significant *P* < 0.05.

GMFI: the geometric mean fluorescence intensity.

DFI: DNA fragmentation index.

**Table 2:** Pre- and postvaricocelectomy semen parameters, sperm DFI, and GMFI of sperm DNA chromatin condensation by flow cytometry.

Variables	Before varicocelectomy	After varicocelectomy	*P* value
Sperm count (mil/mL)	26.2 ± 2.7^*∗*^	51 ± 4.2	0.05
Motility A + B (%)	27.8 ± 16.5	32 ± 7	NS
Normal forms (%)	59.8 ± 13.6	65.03 ± 8.0	NS
Sperm DFI (%) by acridine orange	32.4 ± 7.4^*∗*^	20 ± 4.1	0.05
GMFI of sperm DNA chromatin condensation by flow cytometry (%)	25.4 ± 8.8	22 ± 4.1	NS

^*∗*^Significant *P* < 0.05.

**Table 3:** Comparison between those who achieved clinical pregnancy and those who failed after varicocelectomy.

Variables	Positive pregnancy after varicocelectomy (*n* = 15)	No pregnancy after varicocelectomy (*n* = 60)	*P* value
Sperm count (mil/mL)	59.4 ± 5.3^*∗*^	41 ± 4.2	0.04
Motility A + B (%)	40.2 ± 17.5^*∗*^	22.4 ± 8.4	0.03
Normal forms (%)	67.02 ± 15.4	63.46 ± 11.0	NS
Sperm DFI (%) by acridine orange	16.4 ± 6.4^*∗*^	24.2 ± 4.1	0.04
GMFI of sperm DNA chromatin condensation by flow cytometry (%)	20.3 ± 6.8	23.5 ± 5.4	NS

^*∗*^Significant *P* < 0.05.
